# A large outbreak of measles in the West Midlands, England, 2017–2018: descriptive epidemiology, control measures and lessons learnt

**DOI:** 10.1017/S0950268821000868

**Published:** 2021-04-19

**Authors:** R. Mulchandani, B. Sibal, A. Phillips, S. Suleman, A. Banerjee, R. Teagle, S. Foulkes, K. Spence, O. Edeghere

**Affiliations:** 1 UK Field Epidemiology Training Programme (UK-FETP), Public Health England, London, UK; 2 Field Service Midlands, National Infection Service, Public Health England, Birmingham, UK; 3 National Port Health Team, Public Health England, London, UK; 4 West Midlands Health Protection Team, Public Health England, Birmingham, UK; 5 Midlands Screening and Immunisation Team, Public Health England, Birmingham, UK

**Keywords:** Immunisation, investigation, measles, MMR, outbreak, transmission

## Abstract

In November 2017, eight confirmed measles cases were reported to Public Health England from a hospital in the West Midlands. A multidisciplinary Incident Management Team (IMT) was established to determine the extent of the problem and coordinate an outbreak response. Between 1 November 2017 and 4 June 2018, a total of 116 confirmed and 21 likely measles cases were linked to this outbreak; just under half (43%) were aged over 15 years of age. Fifty-five of the confirmed cases were hospitalised (48%) and no deaths were reported. At the start of the outbreak, cases were mostly individuals of Romanian origin; the outbreak subsequently spread to the wider population. Over the 8-month response, the IMT conducted the following control measures: extensive contact tracing, immediate provision of post-exposure prophylaxis, community engagement amongst specific high-risk groups, MMR awareness raising including catch-up campaigns and enhanced vaccination services at selected GP surgeries. Key challenges to the effective control measures included language difficulties limiting community engagement; delays in diagnosis, notification and appropriate isolation of cases; limited resources for contact tracing across multiple high-risk settings (including GPs and hospitals) and lack of timely data on vaccine coverage in sub-groups of the population to guide public health action.

## Introduction

Measles is a systemic viral infection caused by paramyxovirus, transmitted from person to person by direct contact with nose and throat secretions, or respiratory droplets [1]. In most cases, measles presents as a mild and self-limiting condition with its main features including fever, rash and respiratory illness. However, complications can be severe, particularly for the immunocompromised, pregnant women and younger infants, and include pneumonitis, encephalitis and secondary bacterial infections (including acute otitis media and pneumonia) [1].

The measles, mumps, rubella (MMR) vaccination is the most effective way to prevent measles infection [2]. In the UK, two doses are recommended at around 12 months (MMR1) and 3 years 4 months (MMR2); together, two doses are at least 95% effective in preventing clinical measles [3]. The effectiveness of post-exposure prophylaxis for at-risk groups such as immunocompromised and pregnant women is limited, however it may be advised on a case-by-case basis [4]. Following the introduction of the MMR vaccine in the UK in 1988, measles cases were uncommon, and in 2017, the World Health Organization announced that the UK had achieved measles elimination status, having met the target of ‘sustained interruption of endemic transmission for at least 36 months’; however, this was subsequently lost in 2019 due to an increase in the number of cases detected.

MMR uptake remained high nationally; in 2016–2017, MMR1 and MMR2 uptake was 95.0% and 87.6% across England, respectively [5]. However, inequalities in uptake have been observed by ethnicity, deprivation and geography, and measles cases and outbreaks have often occured disproportionately in under-vaccinated pockets of the population [6, 7].

Throughout 2016 and 2017, large outbreaks of measles were observed across Europe, with particularly high numbers of cases seen in Romania, Italy and Greece mostly amongst unvaccinated or partially vaccinated individuals [8]. In addition, outbreaks were observed across England (including Leeds, Liverpool and Manchester), and led to the subsequent declaration of a national incident at the end of 2017. One of the largest of these outbreaks was in the West Midlands region of England.

This paper describes the key epidemiological findings from the investigation of the outbreak in West Midlands and the public health challenges encountered during the response to the outbreak.

## Methods

### Setting

The West Midlands is a region of central England, comprised of 14 Upper Tier Local Authorities (UTLA), and includes Birmingham, its major city (population estimated at 1.1 million). In 2018, the overall population size of the West Midlands region was estimated at 5.9 million [9].

In 2016–2017, MMR uptake for one dose (MMR1 only) and two doses (MMR1 and MMR2 measured at 5 years of age) across the West Midlands region was estimated at 96.7% and 89.9%, respectively; while in Birmingham, uptake was estimated at 94.9% and 82.9%, respectively [5] (Table 1).

**Table 1.** MMR uptake (%)^a^ by Upper Tier Local Authority (UTLA) in West Midlands, from 2016 to 2020 (Source: COVER^b^, NHS Digital)

	Year			
Local authority	2016–2017	2017–2018	2018–2019	2019–2020
Birmingham	82.9	87.6	82.3	81.4
Coventry	91.7	83.3	81.5	77.9
Dudley	94.8	93.0	93.3	92.2
Herefordshire	93.9	90.4	85.3	88.2
Sandwell	91.1	86.9	85.8	86.3
Shropshire	92.8	88.4	90.3	90.7
Solihull	93.3	88.8	88.5	88.8
Staffordshire	91.2	90.5	90.9	90.1
Stoke-on-Trent	92.9	90.7	88.6	88.3
Telford and Wrekin	92.5	88.3	87.5	87.5
Walsall	88.2	89.5	89.9	88.0
Warwickshire	95.9	90.9	88.1	87.8
Wolverhampton	87.0	87.2	85.4	83.3
Worcestershire	92.4	92.2	87.8	88.8
**West Midlands (region)**	**89**.**9**	**87**.**2**	**86**.**7**	**86**.**1**

a Two dose (MMR1 and MMR2) at 5 years.

b Cover of Vaccination Evaluated Rapidly (COVER).

### Data analysis and case definition

Data on notified measles cases are routinely entered by the West Midlands Health Protection Team (HPT) into HPZone, Public Health England (PHE)'s case management system. HPZone captures demographic, clinical, laboratory and epidemiological data on cases and was updated daily throughout the outbreak. Field Epidemiology Service (FES) maintained a linelist of outbreak cases using the data from HPZone and Second-Generation Surveillance System (SGSS), PHE's microbiology database. Additional information was obtained from Incident Management Team (IMT) notes and from a structured PHE de-brief that took place on 31 July 2018.

The IMT agreed on a definition for confirmed, likely and unlikely cases as outlined below. All cases were then managed using the PHE National Measles Guidance [6].

- **Confirmed case of measles:** A case with clinically typical illness* and laboratory confirmation of acute infection in the absence of recent vaccination or confirmed wild-type measles RNA in any clinical specimen since 01 November 2017 in the West Midlands.
- **Likely case of measles:** A clinically typical* case of measles with epidemiological features that either increase the likelihood of the patient having been exposed and/or favour the diagnosis of measles [6] relative to other causes of febrile illness with symptom onset from 01 November 2017 in the West Midlands.
- **Unlikely case of measles:** A suspected case of measles which does not meet the definition of a likely case, either because it is clinically atypical or because the epidemiological context is not suggestive of measles with symptom onset from 01 November 2017 in the West Midlands.

*Clinically typical measles is defined as measles presenting with classical symptoms, at the minimum cough AND coryzal symptoms AND conjunctivitis AND fever ⩾39 °C in the absence of antipyretics AND maculopapular rash.

Immunisation history was obtained from GP records and Child Health Information System (CHIS) and status was assigned based on the routine NHS schedule. Unvaccinated children under the age of 13 months were categorised as ‘too young’, and those who had received both doses by age five were categorised as ‘fully vaccinated’. MMR % uptake data at UTLA level were obtained from Cover of Vaccination Evaluated Rapidly (COVER) via NHS Digital [5].

Descriptive analyses of the morbidity and immunisation data were performed using statistical package R version 3.5.1. Summary disease frequency statistics were reported as counts, proportions and medians for a range of categorical and continuous variables. Maps showing the spatial distribution of cases were produced using ArcGIS 10.5.1^®^. Cases were mapped against two dose (MMR1 and MMR2) uptake by the fifth birthday, by UTLA.

### Microbiological investigation

Microbiological data from serology and PCR (polymerase chain reaction) testing of throat swabs undertaken by the local NHS laboratories on suspected cases of measles were obtained. Additionally, oral fluid kits were provided by the HPT to all locally confirmed, likely and unlikely cases of measles (regardless of other testing), to enable confirmatory testing and genotyping. Local samples were also sent for confirmatory testing and genotyping at the national PHE reference laboratory.

## Results

### Outbreak notification and initial response

**Fig. 1.**
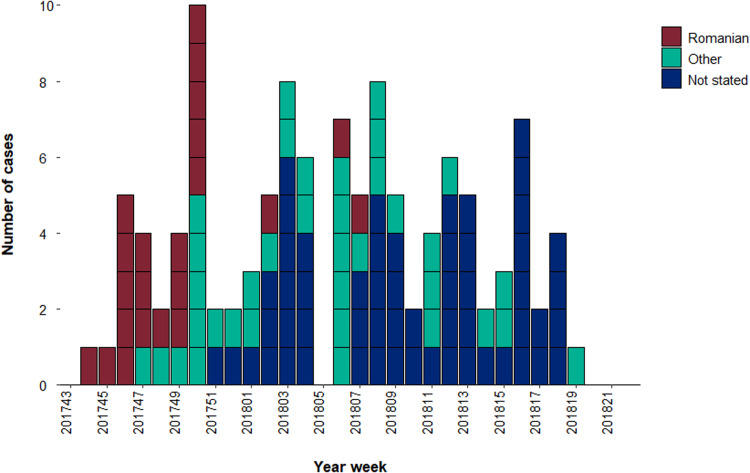
Temporal distribution of confirmed measles cases by case country of origin reported in the West Midlands, 1 November 2017 to 4 June 2018 (Source: HPZone). *Note*: Dates of onset could not be ascertained for three confirmed cases, therefore they are not included in the graph.

On 24 November 2017, the West Midlands HPT were notified by a local National Health Service (NHS) hospital laboratory of a measles PCR-positive throat swab from a child who had been admitted to hospital, with a date of onset on 11 November. By 27 November, an additional seven laboratory-confirmed cases were reported from the same hospital with dates of onset between 16 and 24 November. All were of Romanian origin with recent travel to Romania (3 weeks prior) and resided in the same geographic area of West Midlands; two of the cases were family members of the index case. A majority of cases from November and December had some contact (varying lengths of admission, attending A&E or visiting in-patients) with this local hospital (Fig. 1).

From mid-December, the number of cases continued to increase, with the outbreak spreading to other clinical and non-clinical settings, ethnic groups and geographical areas in West Midlands, beyond the initial geographical and familial clusters. The first local IMT meeting led by the HPT was held on 24 November 2017; five further meetings were held between that date and 31 January 2018.

### Descriptive epidemiology

Between 1 November 2017 and 4 June 2018, a total of 116 confirmed and 21 likely measles cases were linked to this outbreak. Of the confirmed cases, 61 (53%) were male and 55 (47%) were female. Just over half of the cases (n=66; 57%) were children aged <1 to 15 years (40% of all cases were aged <1 to 4 years) (Table 1). The median age was 12.5 years, ranging from 3 months to 55 years. Among confirmed cases, 56 (48%) were admitted to hospital; median age of admitted cases was 4.5 years (range 4 months to 50 years) (Table 2).

**Table 2.** Selected characteristics of confirmed cases of measles reported during the outbreak, West Midlands, 01 November 2017 to 04 June 2018 (Source: HPZone)

Variable	Total (*n* = 116)	%
Age group (years)		
<1	16	13.8
1–4	30	25.9
5–15	20	17.2
16–24	29	25.0
25–39	16	13.8
⩾40	5	4.3
Sex		
Male	61	52.6
Female	55	47.4
Ethnicity		
Eastern European: Romanian	21	18.1
White British	14	12.1
Other^a^	12	10.3
Not stated	69	59.5
Hospitalised		
Yes	56	48.3
No	60	51.7
MMR vaccination status		
Fully vaccinated (age appropriate)	22	19.0
Partially vaccinated	9	7.8
Too young	21	18.1
Not vaccinated	36	31.0
Unknown	28	24.1

a Other includes Asian: Pakistan, Black African, Eastern European: Polish, Eastern European: Czech, White Other, White, Black Caribbean.

**Fig. 2.**
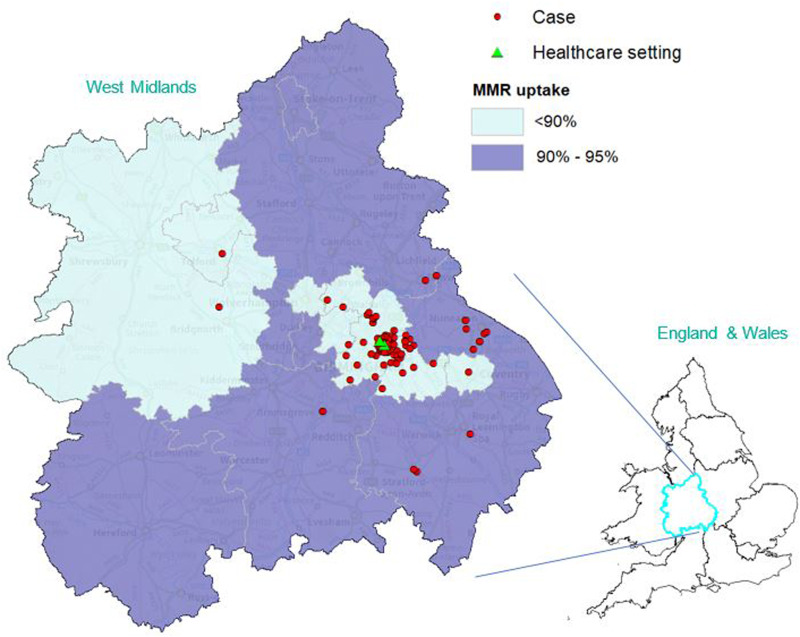
Geographical distribution of confirmed cases of measles reported in West Midlands between 1 November 2017 and 4 June 2018 against the percentage of children vaccinated by their fifth birthday, 2017–18 (Source: HPZone and NHS Digital). *Note*: Healthcare setting refers to the hospital and GP practice associated with the index case at the epicentre of the outbreak.

Twenty-one (18%) of the cases were of known Romanian origin and were mostly reported at the start of the outbreak (the last date of onset for a case known to be of Romanian origin was 8 February 2018) (Table 1; Fig. 1). The largest proportion of confirmed cases resided in Birmingham (n=75; 65%), followed by Solihull (n=18; 16%) and Warwickshire (n=15; 13%) local authority areas; the remaining eight cases were spread across the West Midlands (Fig. 2).

Vaccination status data were available for 88 (76%) of the confirmed cases. Of these, 36 (31%) had not been vaccinated, while 22 (19%) had been fully vaccinated (Table 1). There was very minimal genotypic heterogeneity observed among the cases; 96% (71/74) of typed cases were genotype B3, while three cases (without any known epidemiological links) were genotype D8 (with dates of report between February and May 2018).

### Outbreak management and control

The response to the outbreak was coordinated at three operational levels – (1) PHE West Midlands Centre, supported by (2) PHE National and NHS England, and (3) PHE specialised sub-groups on MMR catch-up and community engagement.

The PHE Centre response was chaired by the West Midlands HPT Consultant in Communicable Diseases (CCDC), with support from virologists, FES, local hospital Infection, Prevention and Control (IPC), and local authority. Their work primarily focused on contact tracing and offering MMR vaccinations and post-exposure immunoglobulins to contacts. The PHE National outbreak response was led by the National Infection Service (NIS), to coordinate and align outbreak response activities across the country. The specialised sub-group consisted of local screening and immunisation team, Local Authority and NHS England, and focused primarily on improving MMR uptake (leading on immunisation strategy) and community engagement (particularly amongst the Romanian community).

Over the course of the outbreak, there were numerous clinical and non-clinical settings attended by cases during their infectious period and which were subsequently targeted for contact tracing; these included nurseries/schools, hospital wards, A&E departments, GP practices and GP out-of-hours waiting rooms and a large local factory. In addition, four confirmed cases had been on international flights during their infectious period. Contact tracing was managed in the community by the HPT and in the hospital by IPC teams.

Management of contacts included ‘warn and inform’ letters encouraging vaccination, measles immunity testing and post-exposure prophylaxis (immunoglobulin), prioritizing vulnerable and unimmunised individuals (including children under the age of one and pregnant women). For hospital staff, MMR status was reviewed by IPC teams and vaccination offered to those with none or incomplete vaccination history to prevent nosocomial transmission.

With a predominance of cases amongst individuals with Romanian origins, MMR vaccination campaigns focused on school and GP settings where there were a known high number of Romanians registered. Initially, three GP practices and four schools were identified for such targeted MMR vaccination campaigns. Across the four schools, a total of 97 children received the MMR vaccination. MMR immunisation through a domiciliary service was also provided to target households with known cases, with limited success. In addition, a time-limited GP-enhanced scheme was established from January 2018 which provided financial compensation to GPs for identifying unvaccinated individuals and proactively contacting them and providing MMR vaccination (primarily with 28 practices but extended to a total of 71). This scheme resulted in 1595 children being immunised, which made up ~17% of all those who had been identified as having no MMR record.

A series of regular and *ad-hoc* communications were sent out to the public and local stakeholders. Written communication was sent to all GPs, acute trusts, ambulance services, GP out-of-hours clinics and schools/nurseries in the West Midlands to increase awareness of MMR vaccination and identification and notification of suspected cases. Communication with the public included proactive media statements in several local newspapers, local television and radio interviews, as well as distribution of leaflets and posters (in both Romanian and English where appropriate).

## Discussion

The findings from the outbreak investigation indicate that the outbreak was likely due to the introduction of measles virus (genotype B3) from Romania, where an outbreak caused by the same strain of measles virus was ongoing [8, 10] (as well as across mainland Europe [11–13], Wales [14] and other Roma populations [15, 16]); and for which previous introductions to the UK have been reported [17]. The outbreak subsequently spread across the West Midlands through pockets of susceptible populations, with the majority of cases occurring in Birmingham (65%). The management of the outbreak presented challenges and we learned several lessons for the prevention and management of similar outbreaks in the future.

In the early stages of the outbreak response, the control measures deployed may have had minimal impact: this wasdue to a lack of local support services engaged specifically with the Romanian community which limited our ability to engage and communicate effectively with the community. In addition, there was some evidence that a proportion of the early cases were from the Roma community, a sub-group who are known to have a lower probability of being vaccinated and lower engagement with health services [18]; however, due to limited language capacity and lack of initial engagement with the health protection team, it was not possible to ascertain in all cases whether those of Romanian descent were part of the Roma community, which subsequently impacted on the quality of information obtained from cases.

Initially, there were challenges in the immunisation activities as it was difficult to identify the ‘pockets’ of lower vaccine uptake within the population, due to a lack of available data. Additionally, due to lower engagement with public services by Romanian and Roma populations (such as GP registration, children's centres and school attendance), language and literacy barriers and a lack of trust in health services [19], there were limited opportunities for providers to communicate the importance of vaccination (and to challenge misinformation), and to invite individuals to immunisation clinics. Together, these highlight the importance of access to timely GP in and out-of-hours community translation services to facilitate the provision of time-sensitive advice on exclusion and contact tracing of cases. Additionally, it emphasises the importance of public sector organisations maintaining good engagement with minority community groups in the area, and recognition of the differences between nationality (Romanian) and ethnicity (Roma), to promote inclusion, understand cultural behaviours and support outbreak control activities in the future.

From the review of IMT minutes and communications with partners, it was evident that roles and responsibilities for information gathering and contact tracing, particularly in some GP practices and hospitals were often unclear; for instance, lack of clarity around specific guidelines for isolation of individuals presenting with a rash in GP and hospital waiting rooms. In addition, there was often a reluctance by some GPs to conduct contact tracing, due to the significant resource implications. Lack of staff resource to check staff immunity records, alongside sub-optimal record keeping, also limited appropriate recommendations for vaccination in healthcare workers, to reduce the possibility of nosocomial transmission. Therefore, it would be important to ensure structures are in place for providing surge capacity across different organisations when required during an outbreak response.

Finally, the reference laboratory did not receive oral fluid samples from 17% of the cases, resulting in a lack of confirmatory testing and genotyping, an important component of measles surveillance required for countries with measles elimination status. Some confusion was identified regarding the need to complete the swab in addition to local testing. Therefore, it would be important to re-iterate the importance of adequately completing an oral fluid kit with all cases (irrespective of any other testing), through further education of clinical staff and having instructions made available in a range of different languages.

Many activities went well in this response and should be applied in future outbreak response. First, there was careful oversight and communications on the progress on the control measures at a local level, through daily incident meetings led by the CCDC; this was complemented by national coordination, which ensured consistency in control measures nationally. Second, a robust system was in place whereby local, regional and national laboratories received the serological or saliva samples, and results were communicated daily to FES and HPT teams (facilitated through SGSS and HPZone systems); this, together with additional resources, ensured efficient and timely contact tracing was conducted. Finally, based on a detailed health needs assessment conducted by West Midlands FES and HPT following the outbreak, the West Midlands' ‘Measles Elimination Board’ and West Midlands' ‘Measles Elimination Plan’ were set up in 2018/2019 to further support measles elimination across the Midlands.

In conclusion, this challenging and protracted measles outbreak in West Midlands was resolved through effective work done through multi-agency engagement. Several areas for improvement were identified following the outbreak [20]. These include improving the identification and communication with hard-to-reach, under-vaccinated local communities; clarifying roles and responsibilities in response teams; increasing awareness amongst GPs of measles guidance, case management and IPC procedures in healthcare settings [21], and improving the availability of vaccine coverage data in sub-groups of the population to allow for targeted public health action in responding to and preventing future outbreaks.

Additionally, several policies could be considered in preventing future outbreaks; for instance, vaccination status checked at the time of school entry to facilitate the offer of MMR (this is not currently in place in the UK), strengthening healthcare worker vaccination requirements, using available data to identify pockets of low vaccination coverage and proactively engaging with the affected groups.

## Data

To request access to any of the data used in the manuscript, please contact the Office for Data Release at Public Health England (odr@phe.gov.uk). Only non-patient identifiable data is available for request.
